# Research progress on the application of feed additives in ruminal methane emission reduction: a review

**DOI:** 10.7717/peerj.11151

**Published:** 2021-03-31

**Authors:** Kang Sun, Huihui Liu, Huiyu Fan, Ting Liu, Chen Zheng

**Affiliations:** College of Animal Science and Technology, Gansu Agricultural University, Lanzhou, China

**Keywords:** Methane, Nitrogenous compound, Plant extract, Prebiotic, Probiotic, Reduction, Ruminant

## Abstract

**Background:**

Ruminal methane (CH_4_) emissions from ruminants not only pollute the environment and exacerbate the greenhouse effect, but also cause animal energy losses and low production efficiency. Consequently, it is necessary to find ways of reducing methane emissions in ruminants. Studies have reported that feed additives such as nitrogen-containing compounds, probiotics, prebiotics, and plant extracts significantly reduce ruminant methane; however, systematic reviews of such studies are lacking. The present article summarizes research over the past five years on the effects of nitrogen-containing compounds, probiotics, probiotics, and plant extracts on methane emissions in ruminants. The paper could provide theoretical support and guide future research in animal production and global warming mitigation.

**Methods:**

This review uses the Web of Science database to search keywords related to ruminants and methane reduction in the past five years, and uses Sci-Hub, PubMed, etc. as auxiliary searchers. Read, filter, list, and summarize all the retrieved documents, and finally complete this article.

**Results:**

Most of the extracts can not only significantly reduce CH_4_ greenhouse gas emissions, but they will not cause negative effects on animal and human health either. Therefore, this article reviews the mechanisms of CH_4_ production in ruminants and the application and effects of N-containing compounds, probiotics, prebiotics, and plant extracts on CH_4_ emission reduction in ruminants based on published studies over the past 5 years.

**Conclusion:**

Our review provides a theoretical basis for future research and the application of feed additives in ruminant CH_4_ emission reduction activities.

## Introduction

Methane (CH_4_) is the world’s second most abundant greenhouse gas after carbon dioxide (CO_2_), accounting for 16% of total greenhouse gas emissions (De Visscher & Van Cleemput, 2003). The potential global warming effect of CH_4_ is 28-fold higher than that of CO_2_ (Stocker et al., 2013). In addition, rumen CH_4_ emissions from ruminants account for 13% to 19% of the global CH_4_ emissions (Liu & Whitman, 2008); therefore, ruminant feeding is a major factor in exacerbating global warming. Therefore, reducing rumen CH_4_ emissions could decrease the rate of global warming, which would be of great significance to efforts to reduce global greenhouse gas emissions. CH_4_ emissions also represent energy losses during ruminant farming. On average, approximately 8–12% of the energy consumed in feed is wasted in the form of CH_4_ emissions (Johnson & Johnson, 1995).

Accordingly, to remedy the low production efficiency and mitigate the potential damage caused by livestock CH_4_ emissions to the environment, researchers have begun to explore the roles of different feed additives in reducing ruminant CH_4_ emissions. Among them, N_2_-containing compounds, probiotics, prebiotics, and plant extracts, which are feed additives that are not harmful to animal health, have been the first subjects of research and are expected to become ideal CH_4_ inhibitors in the future. This article reviews the mechanism of CH_4_ emission production in ruminants and the potential influence of nitrogenous compounds, probiotics, prebiotics, and plant extracts on ruminal CH_4_ production.

## Survey Methodology

In this review, keywords related to additives, ruminants, methane emission reduction in the past five years were searched through the Web of Science database, and Sci-Hub, PubMed, etc. were used as auxiliary searchers. Perform a rough reading of all the retrieved documents; screen out documents related to the effects of additives on ruminant methane in ruminants; then list the documents related to the effects of different additives on ruminant methane in ruminants according to the type of additives; finally, classify different categories Make a summary and finally complete this article.

## Results

### Methane production mechanism in ruminants

**Figure 1:**
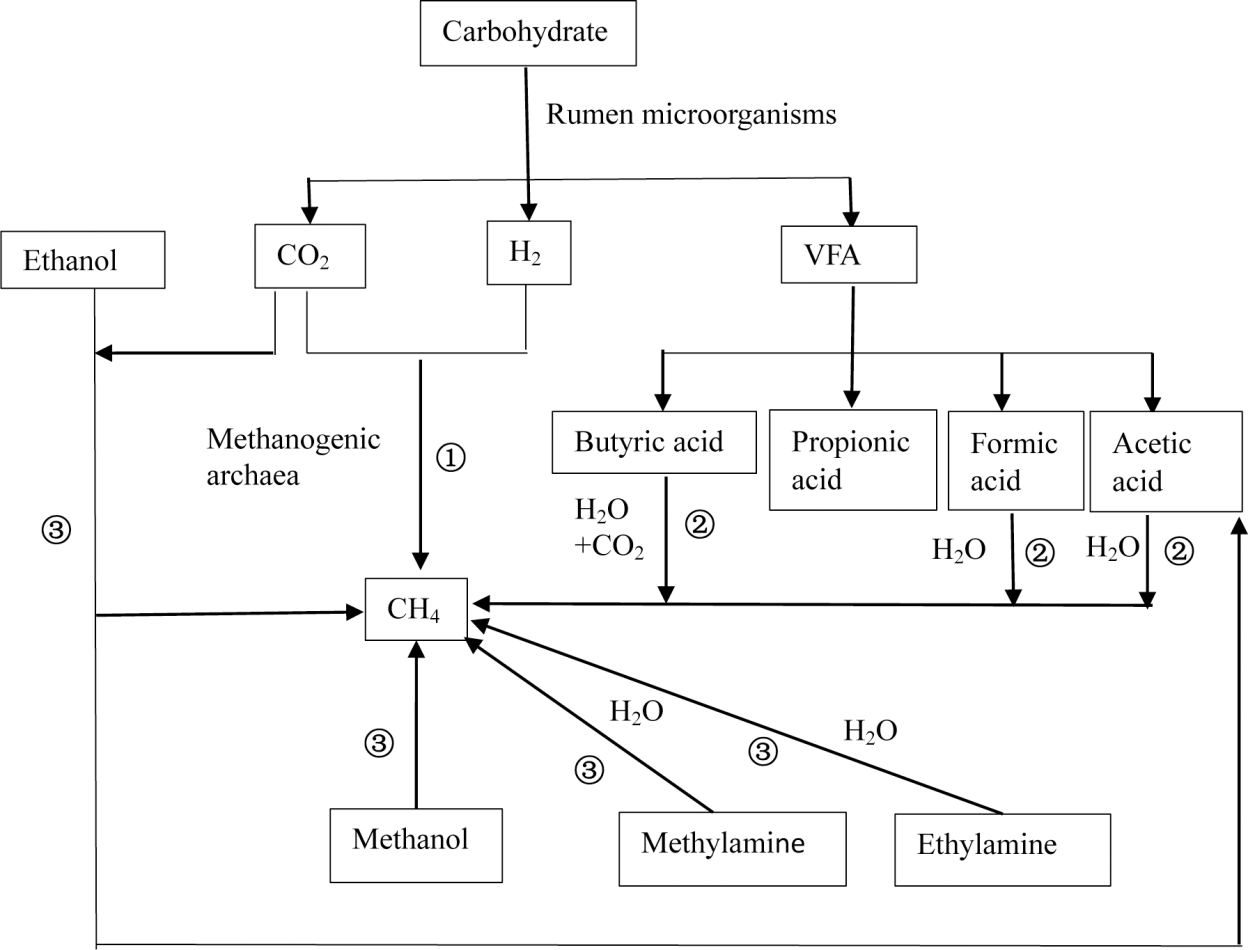
Schematic diagram of methane production. There are three basic pathways of ruminal methane production: (1) represents the CO_2_-H_2_ reduction pathway, (2) represents the synthesis pathway of short chain fatty acids such as formic acid, acetic acid, and butyric acid as substrates, and (3) represents the synthesis pathway with methyl compounds such as methanol and ethanol as substrates. Among these, route (1) is considered to be the primary route of methane production.

After ruminant ingestion, the nutrients (proteins, lipids, and carbohydrates) in feed are degraded by rumen microorganisms to produce hydrogen (H_2_) and primary fermentation products that contain methyl groups such as formic acid, acetic acid, methanol, and methylamine. Afterward, methanogens convert the primary fermentation products into CH_4_ and energy is obtained. There are three pathways of ruminal CH_4_ production (Thauer et al., 2008) (Fig. 1), including (1) the CO_2_-H_2_ reduction pathway; (2) synthesis pathways using short-chain fatty acids such as formic acid, acetic acid, and butyric acid as substrates; (3) and synthesis pathways using methyl compounds such as methanol and ethanol as substrates. Among the three, the CO_2_–H_2_ route is the primary pathway (Ellis et al., 2008) because the growth rates of *Methanococcus* that exploit acetic acid are low (Liu & Whitman, 2008), and acetic acid-producing bacteria have a low affinity for H_2_ (Morgavi et al., 2010). In addition, only methanogens of *Methanosphaera* use methanol to produce CH_4_ (Liu & Whitman, 2008).

### Effects of nitrogenous compounds on methane production in ruminants

N-containing compounds are used as ammonium-N (NH_4_^+^-N) supplements in ruminant diets. Extensive research has revealed that N-containing compounds can reduce CH_4_ production via their influence on rumen microorganisms, for example, by reducing the activity of participating CH_4_-producing enzymes and competing for hydrogen (Table 1), in addition to supplementing NH_4_^+^-N. Among them, nitrate-N (NO^−^_3_-N) is considered to be a urea substitute; it can not only meet the requirements of rumen microorganisms for NH_4_^+^-N, but can also decrease CH_4_ production substantially (Adejoro et al., 2020; Adejoro, Hassen & Thantsha, 2018; Alvarez-Hess et al., 2019; Wu et al., 2019). The mechanism of action is linked to the competitive effects of NO^−^_3_-N over H_2_ consumption and the inhibitory effect of the generated nitrite (NO^−^_2_-N) on methanogen proliferation. However, large doses of NO^−^_3_-N may cause the accumulation of toxic NO^−^_2_-N (Jeyanathan, Martin & Morgavi, 2014). Therefore, it is necessary to control NO^−^_3_-N dosages, or supplement feed with NO^−^_2_-N reducing agents, to minimize nitrite toxicity (Jeyanathan, Martin & Morgavi, 2014); NO^−^_2_-N capsules could also be used (De Raphelis-Soissan et al., 2017).

**Table 1:** Inhibitory effects of nitrogen-containing compounds on ruminal methane emissions and their mechanisms.

Types of nitrogenous compounds	Inhibitory effect	Addition amount; maximum methane suppression amount	Inhibition mechanism	References
Nitrate	^***^	20 mg/g dry matter; 21% (Alvarez-Hess et al., 2019) 5 mmol/L; 32.92% (Wu et al., 2019) 5 mM; 43.26% Liu et al., 2017)	(1) Hydrogen consumption; (2) Inhibits the proliferation of methanogens; reduces their activity and abundance	Adejoro et al. (2020); Adejoro, Hassen & Thantsha (2018); Alvarez-Hess et al. (2019); Wu et al. (2019); Liu et al. (2017); Zhao et al. (2018)
Encapsulated nitrate (EN)	^**^	70 g /100 kg of body weight; 18.5% CH_4_/kg of forage dry matter intake (Granja-Salcedo et al., 2019) 2.5%; 9.37 mM/d (Capelari et al., 2018) 2.5%; 2.8 g/kg Dry matter intake (Alemu et al., 2019)	Reduces methane reducing bacteria	Granja-Salcedo et al. (2019); Capelari et al. (2018); Alemu et al. (2019)
Urea and nitrate mixture	^**^	34 g/kg straw dry matter + 6 g/kg dry matter of ammonium nitrate; 10.2% (Zhang et al., 2019) Urea + ammonium nitrate (34 + 6 g/kg of dry matter, respectively); 3.1 mL/g dry matter (Zhang et al., 2018)	Indirect consumption of hydrogen	Zhang et al. (2019); Zhang et al. (2018)
Nitroethane (NE), 2-Nitroethanol (NEOH), 2-Nitro-1-Propanol (NPOH)	^***^	10 mmol/L; 96.7% (NE), 96.7% (NEOH), 41.7% (NPOH)	(1) Inhibits the activity of methanogens; (2) Inhibits methyl-coenzyme M gene expression; (3) Reduces the content of coenzyme F420 and F430	Zhang et al. (2020a)
3-Nitrooxypropanol (NOP)	^***^	0.08 mg/g dry matter; 44% (Alvarez-Hess et al., 2019) 2.5 g/animal/day; 38%/kg dry matter intake (Martinez-Fernandez et al., 2018) 60 mg/kg of feed dry matter; 26%/day (Melgar et al., 2020) 1.6 g; 28%(roughage), 23% (concentrate pellet) (Van Wesemael et al., 2019)	Inhibits methyl-Coenzyme M activity	Alvarez-Hess et al. (2019); Dijkstra et al. (2018); Kim et al. (2020a); Martinez-Fernandez et al. (2018); Melgar et al. (2020); Van Wesemael et al. (2019); Jayanegara et al. (2018); Henderson, Cook & Ronimus (2018)

**Notes.**

*** The additive has a significant effect on methane inhibition.

** The additive has a general effect on methane inhibition.

A novel N-containing compound, 3-nitrooxypropanol (3-NOP), has recently been introduced; it can continuously reduce CH_4_ production without adversely affecting animal growth or development (Romero-Pérez et al., 2016). It is an ideal CH_4_ inhibitor. The structure of 3-NOP is similar to that of methyl-coenzyme M, which is associated with the last step of CH_4_ production, and 3-NOP can inhibit the activity of the reductase. Nitroethane (NE), 2-nitroethanol (NEOH), and 2-nitro-1-propanol (NPOH) can also inhibit methanogenic bacteria and significantly reduce the expression of the methyl-coenzyme M reductase gene (Zhang et al., 2020a). In addition, these compounds could reduce the content of coenzymes F420 and F430, reducing ruminal CH_4_production in turn (Zhang et al., 2020a). There are numerous other N-containing compounds that inhibit methanogen activity and alter the structure of rumen microbial flora, the activity of enzymes involved in CH_4_ production, and the distributions of volatile fatty acids, leading to the consumption of H_2_ and reduction of CH_4_ production in turn.

### Effects of probiotics on methane production in ruminants

Probiotics are a class of beneficial active microorganisms or their cultures. Probiotics could reduce CH_4_ emissions in ruminants (Table 2). There are many types of probiotics, and different strains have different inhibitory effects on CH_4_ emissions. For example, the GA03 strain of *Acetobacter* is more effective at inhibiting CH_4_ production than other isolated strains (Kim et al., 2020b). Most probiotics reduce CH_4_ production by influencing the activities of ruminal microorganisms, with no adverse effects on animals. In addition, probiotics enhance ruminal fermentation.

**Table 2:** The inhibitory effects of probiotics on ruminal methane emissions and their mechanisms.

Types of probiotics	Inhibitory effect	Addition amount; maximum suppression methane amount	Inhibition mechanism	References
Propionic acid bacillus	*(Most propionic bacteria) ^***^(*P. jensenii* LMGT282 and *P. thoenii* LMGT2827 or T159)	100 µL of the *propionic acid bacteria* culture (2 × 10^8^ to 4 ×10^8^ colony forming units), *Propionibacterium thoenii* T159; 20%	Unknown	Chen et al. (2020)
Lactic acid bacteria	^***^	5.3 lg cfu/g fresh weight, *Lactobacillus plantarum*, 8.8 ml/g(72h)	Hydrogen consumption	Guo et al. (2020)
Acetic acid bacteria	^***^	1% *Proteiniphilum acetatigenes* GA03; -	Reduced the number of methanogens	Kim et al. (2018c); Kim et al. (2020b)
Enterococcus faecium SROD	^***^	0.1%; 2.08 mM/mL	Alters microbial flora	Mamuad et al. (2019)
Probiotic products of *Ruminococcus flavefaciens*	^***^	2 g probiotic products in powder; 1.2 ml/g of dry matter 10 ml probiotic products in liquid; 1.2 ml/g of dry matter	Reduce the number of rumen protozoa	Hassan et al. (2020)
*Bacillus licheniformis*	^***^	2. 5 ×10^9^; 2.7 L/d	Unknown	Deng et al. (2018)
*Saccharomyces cerevisiae*	^**^(Pedraza-Hernández et al., 2019) ^*^ (Darabighane et al., 2019)	–	Affects rumen microbes	Pedraza-Hernández et al. (2019); Darabighane et al. (2019)

**Notes.**

*** The additive has a significant effect on methane inhibition.

** The additive has a general effect on methane inhibition.

* The additive has no obvious effect on methane inhibition.

Lactic acid bacteria, which have been used as feed additives for a long time, not only reduce CH_4_ emissions per unit volatile fatty acid (VFA) output, but also improve the fermentation quality and fiber digestibility of silage (Guo et al., 2020). In addition, the denitrifying bacterium *Bacillus* 79R4 could prevent NO^−^_2_-N poisoning and microbial ecosystems from impairing fermentation efficiency (Latham et al., 2019). Furthermore, *Bacillus licheniformis* reduces CH_4_ production and increases feed energy and protein utilization (Deng et al., 2018). However, the inhibitory mechanism of lactic acid bacteria on CH_4_ is still unclear; therefore, in the future, more research will need to be conducted on the influence of lactic acid bacteria on rumen microbes and hydrogen competition to elucidate the mechanism of inhibiting CH_4_ production.

### Effect of prebiotics on methane production in ruminants

Prebiotics are substances that are not easily digested or absorbed by the host. They selectively stimulate the growth and activity of one or several ruminal microorganisms with a positive effect on ruminal fermentation (De & Schrezenmeir, 2002). Prebiotics suppress ruminal CH_4_ production in ruminants. Prebiotics mainly reduce rumen CH_4_ production by altering the bacterial community structure, influencing the permeability of the cell walls of methanogenic archaea, and stimulating other bacteria to compete with methanogens for H_2_ (Table 3). According to Tong et al. (2020), the prebiotic chitosan can influence bacterial community structures by altering microbial population compositions, for example, by replacing fibrinolytic enzyme-producing microbes (*Firmicutes* and *Fibrobacteres*) with amylolytic enzyme-producing microbes (*Bacteroides* and *Proteus*); in turn, reducing CH_4_ production.

**Table 3:** Inhibitory effects of prebiotics on ruminal methane emissions and their mechanisms.

Types of prebiotics	Inhibitory effects	Addition amount; maximum methane suppression amount	Inhibition mechanism	References
Chitosan	^***^	3000 (molecular weights) dry matter; 22.9% ml/day (Tong et al., 2020) 2% Chitosan + 21% of crude glycerin; 53.67% (Seankamsorn, Cherdthong & Wanapat, 2020)	(1) Alters microbial community structure (Tong et al., 2020) (2) Alters fermentation pathway (Seankamsorn, Cherdthong & Wanapat, 2020) (3) Influences methanogenic bacteria cell wall permeability (Zanferari et al., 2018)	Tong et al. (2020); Seankamsorn, Cherdthong & Wanapat (2020); Zanferari et al. (2018); Haryati et al. (2019)
Yeast products	^***^	4 mg/1 g dry matter; -	Indirect consumption of hydrogen	Vallejo-Hernández et al. (2018)

**Notes.**

*** The additive has a significant effect on methane inhibition.

According to Seankamsorn, Cherdthong & Wanapat (2020), chitosan could influence the ruminal fermentation process by altering VFA distributions and increasing propionic acid concentrations, which reduces CH_4_ production in turn. However, according to some researchers, the reduction in CH_4_ is associated with the degree of chitosan deacetylation, which could alter the permeability of the methanogen cell wall. In addition, Vallejo-Hernández et al. (2018) observed that various yeast products could reduce CH_4_ emissions by stimulating acetic acid-producing bacteria to compete with methanogens or metabolize hydrogen. Overall, compared with other feed additives, prebiotics are still relatively less applied in feed, and their types are limited. Therefore, future research should target strategies to promote the adoption prebiotic feed additives. For example, when conducting scientific research, it is necessary to strengthen contact with breeding companies so that companies can see the effects of prebiotic additives and reduce penalties due to pollution. It is also necessary to enforce the guidelines and requirements of the national environmental protection department for low environmental pollution and exploit consumers’ demand for healthy food to promote the widespread use of prebiotic additives.

### Effects of plant extracts on methane production in ruminants

In recent years, the effects of plant-derived feed additives on rumen microbial fermentation, rumen CH_4_ production, and ruminant performance have been increasingly recognized. Many previous studies have demonstrated that natural plant-derived compounds are promising anti- CH_4_-generation compounds, including tannins, essential oils, and saponins (Table 4). Although plant extracts have potentially significant effects on CH_4_ emission reduction in ruminants, most of the inhibition mechanisms are not clear. According to research findings, the effects of plant-derived feed additives on ruminant methane emission reduction are mainly based on competition for hydrogen and rumen microbes. Competing for hydrogen is manifested in the form of increased propionic acid contents in fermentation products. Effects on rumen microbes are manifested in the number and activity of protozoa, methanogens, and total bacteria, and the results vary based on types of plant-derived feed additives.

**Table 4:** Inhibitory effects of plant extracts on ruminal methane emissions and their mechanisms.

Types of probiotics		Inhibitory effects	Addition amount; maximum suppression methane amount	Inhibition mechanisms	References
Plant extracts	*Corymbia citriodora* leaf extract	^***^	10 ml/calf/day; –	(1) Protozoa number reduced (1. 84 ×10^5^/ml) (2) Ratios of volatile fatty acids altered	Hassan et al. (2020)
	*Aloe vera*, *Carica papaya*, *Azadirachta indica*, *Moringa oleifera*, *Tithonia diversifolia*, *Jatropha curcas*, and *Moringa oleifera* pod extracts	^***^	25 mg/L and 50 mg/L Azadirachta indica, Carica papaya, Tithonia diversifolia; 15%. Jatropha curcas and Moringa oleifera pods; 30% (Akanmu & Hassen, 2018) Eragrostis substrate, 4 ml plant extracts; 50% reduction in methane / Total gas production (Akanmu, Hassen & Adejoro, 2020)	Unknown	Akanmu & Hassen (2018); Akanmu, Hassen & Adejoro (2020); Parra-Garcia et al. (2019)
	Pomegranate peel extract and Desert teak extract	^***^	2% of dry matter intake, Punica granatum; 46% Tecomella undulata; 42%	Unknown	Hundal et al. (2019)
	*Rhus succedanea* extract	^***^	50 mg/L; lowest	Unknown	Kim et al. (2018a)
	*Areca catechu* and *Acacia nilotica* extract	^**^	2% dry matter basis, Areca Catechu; 21%; Acacia nilotica; 23%	Unknown	Wadhwa, Sidhu & Bakshi (2020)
	*Asparagopsis armata*	^***^	1.0%; 67.2% (Roque et al., 2019)	Unknown	Roque et al. (2019); Lee et al. (2018)
	Garlic extract	^***^	0.5%; –(Kim et al., 2018b) 1 g the experimental mixture; 6.9 ±10.7 ml/d (Eger et al., 2018)	Decreased abundance of methanogenic archaea	Kim et al. (2018b); Eger et al. (2018)
	Plant extract; resveratrol	^***^	25 mg, high-forage diets; 60%; high-concentrate diets; 41%	Decreased abundance of Methanobacter	Ma et al. (2020)
	Plant extracts: caffeic acid and p-coumaric acid	^***^	12 mM, Caffeic acid; 37.58%; p-coumaric acid; 28.33%	Unknown	Berchez et al. (2019)
	Licorice extract	^***^	1 g/L; 51%	(1) Decline in the number of rumen protozoa (1.27 log cells/mL) (2) Decrease in bacterial diversity (3) Change in bacterial and archaea community structure	Ramos-Morales et al. (2018)
	Eucalyptus leaf extract	^***^	100 mg ethyl acetate extract; 93.4%	Unknown	Boussaada et al. (2018)
	Ginkgo extract	^***^	1.6% fruit equivalent, Forage-to-concentrate ratio 5:5; 41.9%	(1) Hydrogen consumption (2) Reduce the number of rumen flora (Decrease in total bacteria, *Ruminococcus flavefaciens*, *Ruminococcus albus* and *Fibrobacter succinogenes*. Increase in the levels of *Selenomonas ruminantium*, *Anaerovibrio lipolytica*, *Ruminobacter amylophilus*, *Succinivibrio dextrinosolvens* and *Megasphaera elsdenii*)	Oh, Koike & Kobayashi (2017)
	Olive leaf extract	^***^	In oaten chaff treatments: Leccino leaf chloroform extract; 86.4% Kalamata leaf chloroform extract; 69.9% In commercial concentrate treatments: Leccino leaf chloroform extract; 94.5% Kalamata leaf chloroform extract; 92.5%	Decreased ratio of acetic acid and propionic acid, Hydrogen consumption	Shakeri et al. (2017)
	Radish extract	^***^	12 hr incubation time 5, 7 and 9%; highest methane reduction	Unknown	Lee et al. (2017)
	Propolis extract	^***^	Within 5 h, the methane production decreases linearly	Hydrogen consumption	Santos et al. (2016)
	Malic acid or disodium malate	^***^	Treatment of sunflower meal with malic acid; 11.3% Treat sunflower seeds with malic acid; 15.5%	Propionic acid increases, consumption of hydrogen	Vanegas et al. (2017a); Vanegas et al. (2017b)
	Mulberry leaf flavonoids	^***^	2 g/head/day; 12%	(1) Reduction in the number of methanogens in the rumen; (2) Decline in the number of rumen protozoa (3) Increase in populations of *Fibrobacter succinogenes*, *R. albus* and *Butyrivibrio fibrisolvens*	Ma et al. (2017)
	Saturated medium chain fatty acids	^***^	2.5% Krabok (*Irvingia malayana*) seed oil and *Flemingia* (*Flemingia macrophylla*) leaf powder; Lowest yield	(1) Hydrogen consumption (2) Decrease in the numbers of methanogens and ciliates or inhibition of their activity.	Kang et al (2017); Dohme et al. (2000); Patra (2013)
	Lauric acid	^***^	30 g/kg dry matter; the methane to total gas ratio with the Lauric acid diet was significantly reduced from day 4 onwards, to almost 0 at day 8	1) Hydrogen consumption	Klop et al. (2017)
	Grape pomace Powder	^***^	5.0 kg Dried grape marc dry matter/day; 23% 5.0 kg Ensiled grape marc dry matter/day; 18%	(1) Hydrogen consumption (2) Significant decrease in the number of protozoa and *Cellulolytic bacteria*.	Foiklang, Wanapat & Norrapoke (2016); Moate et al (2014)
Medicinal plant extracts	Honeysuckle extract	^***^	Methane production (ml/g dig dry matter ) decreased linearly with increasing concentrations of the *Honeysuckle extract*	Decrease in the total number of microorganisms, methanogenic archaea, and ciliate protozoa. And at 3%, *Ruminococcus albus*, *Fibrobacter succinogenes*, and *Ruminococcus flavefaciens* decreased significantly.	Yejun et al. (2019)
	Papaya leaf extract	^***^	Methane production (mL/250 mg dry matter ) decreased with increasing levels of Papaya leaf extract.	(1) Hydrogen consumption (2) Decreases in the total bacteria, total protozoa, *Butyrivibrio fibrisolvens* and methanogen populations	Jafari et al. (2018); Jafari et al (2016)
	Bamboo Leaf	^***^	25%; 62%	Hydrogen consumption	Jafari et al. (2020)
	Chicory	^***^	Pure chicory; 23%( when expressed per kg dry matter intake)	Unknown	Niderkorn et al. (2019)
	Patchouli and Atractylodes	^***^	25 g/kg dry matter; Cablin patchouli herb and Amur cork tree abated methane release	(1) Decrease in methanogens, *Ruminococcus flavefaciens*, and total fungi populations (2) Hydrogen consumption	Wang et al. (2019a); Wang et al. (2019b)
	A mixture of absinthe, chamomile, fumigant and sunflower	^**^	Potential to reduce methane emissions from the rumen	Unknown	Petrič et al. (2020)
	Rhubarb	^***^	1 g/d; 14%(García-González, González & López, 2010) 1.33 g/L; 55%(Kim et al., 2016)	(1) Hydrogen consumption (2) Increase in numbers of *Prevotella* and *Lactobacillus*, but decrease in *Methanobrevibacter*. (3) Depletion of methyl-Coenzyme M reductase binding sites	García-González, González & López (2010); Kim et al. (2016); Arokiyaraj, Stalin & Shin (2019)
	*Boerhovia diffusa*, *Holarrhena antidysentericum*, *Solanum nigrum*, *Trigonella foenum-graecum*, *Withania somnifera* and *Woodfordia fruticosa*	^***^	When compared irrespective of the source of inoculum, methane production reduced linearly with the increasing dose of supplementation. Withania somnifera, Woodfordia fruticose and Boerhovia diffusa more effective in reducing methanogenesis;	Unknown	Pattanaik et al. (2018)
	Myrobalan	^***^	Ruminal methane production was linearly decreased with increasing level of *Terminalia chebula* supplementation	(1) Hydrogen consumption (2) Reduction in the number of protozoa	Anantasook et al. (2016)
	Sanguisorba	^***^	40 mg and 100 mg; methane expressed per units of total gas production decreased in a linear and quadratic manner	(1) Decrease in the protozoal population (2) Reduction of in vitro dry matter digestibility	Cieslak et al. (2016)
	*Centella asiatica* powder and Mangosteen peel power	^***^	25 g/kg dry matter intake; 4.8%	(1) Reduction in the number of rumen protozoa (2) Increase in Cellulolytic bacteria, Proteolytic bacteria, and *F. succinogenes*	Norrapoke et al. (2014)
Plant tannins	Rambutan peel	^***^	16 mg; 1.3 mL/0.5 mg dry matter	Hydrogen consumption	Gunun et al. (2018)
	Black wattle bark extract	^***^	30 g Acacia/kg of dietary dry matter; Linear decline, 0.18 g/day or 0.16 g/kg dry matter intake	Unknown	Denninger et al. (2020)
	Acacia leaf	^***^	36% of dry matter; 19.6% (Montoya-Flores et al., 2020) 100% leaves; 55% Rira et al. (2019) 100% pods; 64% (Rira et al., 2019)	Unknown	Montoya-Flores et al. (2020); Rira et al. (2019)
	Pitaya peel powder	^**^	4% of dry matter; roughage to concentrate ratio 100:0; 2.4 mmol/L.70:30;3.8 mmol/L.30:70;2.9 mmol/L	(1) Reduction of the number of rumen protozoa (2) Hydrogen consumption	Matra et al. (2019)
	Chinese chestnut	^**^	1.5 g/day chestnut tannins; 65%	Decrease in methanogens, *Ruminococcus albus*, *Methanobrevibacter sp*., *Methanobrevibacter ruminantium*, and *Methanosphaera stadtmanae*.	(Witzig, Zeder & Rodehutscord, 2018)
	Quebracho tannin	^***^	3%/kg dry matter; 41% ( (Pineiro-Vazquez et al., 2018b)) 4.5% of dry matter; 20.38 L/d (Norris et al., 2020)	Unknown	Pineiro-Vazquez et al. (2018b); Liu et al. (2019a); Norris et al. (2020)
	Red bean grass and Hazelnut peel extract	^**^	15.2% Sainfoin pellets+4.1% hazelnut pericarps; -	Unknown	Niderkorn et al. (2020)
	Tannin-rich peanut skins and wet lees	^***^	20% peanut skin+15% Wet distiller’s grains plus solubles; 0.17 ml/24 h 15% peanut skin+10% Wet distiller’s grains plus solubles; 0.28 ml/24 h	(1) Hydrogen consumption (2) Decrease in the average populations of Bacteroidetes, total methanogens, *Methanobrevibacter sp*. AbM4, and total protozoa.	Min et al. (2019)
	Legumes leaves and pods	^***^	15% of dry matter; 4.7 g/day (Molina-Botero et al., 2019a)	Unknown	Molina-Botero et al. (2019a); Molina-Botero et al. (2019b)
	Grape seed extract	^***^	2 g/kg dry matter; 2.7 mg/day	(1) Hydrogen consumption (2) Significant increase in the relative abundance of *Methanomassiliicoccus*; Significant reduction in the relative abundance of *Methanobrevibacter*. (3) Significant reduction in the number of Ciliate protozoa, and Methanogens; Significant increase in the number of Anaerobic fungi.	Zhang et al. (2020b)
	Mangosteen Peel	^***^	30 mg/500 mg dry matter; 0.549/total gas (Paengkoum Phonmun et al., 2015) 50% Mangosteen peel+50% concentrate; 1.7 ml/ dry matter (Shokryzadan et al., 2016) 25 g/kg dry matter intake; 2.5 (Norrapoke et al., 2014)	(1) Hydrogen consumption (2) Decrease in the number of total protozoa, total methanogens, *Ruminococcus flavefaciensand*, and *Butyrivibrio fibrisolvenes*; Increase in the total number of bacteria, cellulolytic bacteria, Proteolytic bacteria and *F.succinogenes*.	Paengkoum Phonmun et al. (2015); Shokryzadan et al. (2016); Wanapat et al (2014); Norrapoke et al. (2014)
	Contains tannins of sumac, chestnut, oak and mimosa	^**^	0.5,0.75 and 1 mg/ml; decrease linearly with added amount	Decrease in the number of total methanogens, *Ruminococcus flavefaciens*, and *Fibrobacter succinogenes*	Jayanegara et al. (2015)
	Delonix regia seed meal	^***^	16.7 mg of dry matter; 42.4%	Reduction in the number of protozoa	Supapong et al. (2017)
	Banana flower powder pellet	^**^	0, 30, and 60 g/kg of dietary substrate; decrease linearly with added amount	Reduction in the number of protozoa; Increase in number of bacteria	Kang, Wanapat & Viennasay (2016)
Plant essential oil	Lippen and Marigold essential oil	^***^	300 mL/L incubated substrate; day 6 onward > 90%	Unknown	Garcia et al. (2019)
	Patchouli essential oil	^***^	90 µg/g incubated substrate; 9%	Unknown	El-Zaiat & Abdalla (2019)
	Thymol and carvacrol oils	^**^	0.2 g/L bovine ruminal culturemedium, (Castañeda Correa et al., 2019) 100 µL/L; 2.89 mmol per 100 mol VFAs (Baraz, Jahani-Azizabadi & Azizi, 2018)	Unknown	Castañeda Correa et al. (2019); Baraz, Jahani-Azizabadi & Azizi (2018)
	Agolin	^***^	0.05 g/kg dry matter; -	Unknown	Belanche et al. (2020); Klop et al. (2017)
	Oregano essential oil	^***^	52 mg/L; 6.4 ml( 24 h)	Increase in the relative abundance of *Prevotella* and *Dialister* bacteria	Zhou et al. (2020)
	Citrus essential oil	^**^	0.8 mL / L; -	Reduction in rumen microbial adaptability	Wu et al. (2018)
	Microencapsulated blend of essential oil	^***^	200 mg of microencapsulated blend of essential oils/kg dietary dry matter; 13.7/kg digestible organic matter	Unknown	Soltan et al. (2018); Yatoo et al. (2018)
	Moringa seed oil	^***^	Roughage to concentrate ratio 70:30, 4% incubated substrate; 3.29 total CH_4_ mL/g dry matter Roughage to concentrate ratio 50:50, 1% incubated substrate; 2.81 total CH_4_ mL/g dry matter Roughage to concentrate ratio 30:70, 1% incubated substrate; 1.31 total CH_4_ mL/g dry matter	(1) Hydrogen consumption (2) Roughage to concentrate ratio 30:70, 50:50; Reduction in the number of *Firmicutes* to *Bacteroidetes* ratio, protozoa and methanogens. 70:30; Significant increases in the numbers of protozoa, methanogens and bacteria.	Ebeid et al. (2020)
	Eucalyptus oil	^***^	10 ml/kg dry matter, Roughage to concentrate ratio 60:40; 46%	Reduction in the number of rumen protozoa	Abdelrahman et al. (2019); Wang et al. (2018)
	Anise oil	^***^	400 mg/L; 39 mL/g of digestible dry matter	Unknown	Wang et al. (2018)
	Silkworm pupa oil	^***^	5%; 30% (Thirumalaisamy et al., 2020)	Reduction in the number of rumen protozoa	Thirumalaisamy et al. (2020)
	Tucumã oil	^***^	1%, forage:concentrate, 70:30; 0.66 mg/g dry matter	Hydrogen consumption	Ramos et al. (2018)
	Linseed oil	^***^	4%; 17% (Guyader et al., 2015) 4.8 mg/mL; 18% (Ruiz-Gonzalez et al, 2017) 6%; 46 mL/day (Vargas et al., 2020)	(1) Hydrogen consumption (2) Reduced the number of protozoa and copy number of total bacteria	Ruiz-Gonzalez et al (2017); Vargas et al. (2020); Guyader et al. (2015)
Plant saponins	Tea extract	^**^	0.028%, forage-to-concentrate ratio 60:40; 3g/day (Kolling et al., 2018) 2.0 g/head/day; 8.80% (emissions scaled to metabolic body weight) (Liu et al., 2019b)	Unknown	Kolling et al. (2018); Liu et al. (2019b)
	Ivy fruit saponins	^***^	5% dry matter; 1.98 mmol/day	Anaerobic fungi and Methanogens content decreased	Belanche et al. (2016)
Waste products	Humic acid	^***^	3.6 mg/mL; 1.6 mL/g dry matter(48 h)	Unknown	Sheng et al. (2019)
	Geen tea waste	^***^	40 g/kg dry matter; 3.39 ml/200 mg dry matter	Unknown	Nasehi et al. (2018)
	Palm oil industrial waste phospholine gum	^***^	50%; completely inhibited methane production.	(1) Reduced the content of methanogens, Lactoba-cillus sp. and Megasphaera sp. (2) Hydrogen consumption	Sheng et al. (2019); Nasehi et al. (2018); Mustapha et al. (2017)
	Wastes of tomato fruit	^***^	Replacing 50% of cereals-based concentrate; 28%	Hydrogen consumption	Romero-Huelva & Molina-Alcaide (2013)

**Notes.**

*** The additive has a significant inhibitory effect on methane.

** The additive has an inhibitory effect on methane.

Medicinal plant extracts (for example: patchouli, atractylodes, and honeysuckles), tannins, and essential oils have all been shown to suppress the production of CH_4_ by altering ruminal microbial structure and abundance (Kim et al., 2016). The inhibitory effects of tannins on CH_4_ reduction are influenced by their molecular weight (Petlum et al., 2019; Piñeiro Vázquez et al., 2018a). However, if the molecular weights of tannins are too high, the palatability of the diet would be adversely affected, and, in turn, the performance of animals. Therefore, it is critical to determine the optimal tannin supplementation levels. The effects of plant essential oils on rumen microorganisms could be linked to their antibacterial, antiviral, antifungal, and insecticidal properties. Plant essential oils contain various active ingredients that can regulate rumen fermentation and reduce CH_4_ emissions (Soltan et al., 2018). As antibiotic substitutes, medicinal plant extracts could have unique influences on rumen microbes due to their equally unique medicinal properties, including CH_4_ emission reduction (Kim et al., 2016; Yadeghari et al., 2015).

Generally, plant extracts have a significant effect on reducing methane emissions from ruminants, but most of its mechanism of action is still unclear. Almost all tests are in vitro tests, which are short-term tests. At present, research on plant extracts in animals is still lacking, and the effect of long-term use of plant extracts on animals is still unclear. Therefore, in the future, we should focus on using plant extracts in animals and study the effects of long-term use on animals.

## Conclusions

Considering the results of the studies that have been published over the past 5 years, the application of nitrogenous compounds, probiotics, prebiotics, and plant extracts has been shown to reduce ruminal CH_4_ emissions. There are three main ways of reducing CH_4_ production: (1) reducing the number of rumen protozoa and inhibiting methanogen activity; (2) increasing propionic acid production to compete with methanogens for hydrogen; (3) inhibiting the activity of enzymes involved in methanogen activity. However, the mechanisms of action of most plant extracts remain unclear; and almost all studies are based on in vitro fermentation tests. In addition, most plant extracts have no adverse effects on animals, and they are rich in resources. Consequently, research on the effects of plant extracts in animals and their mechanisms of action should be the main research direction in the future, to enhance their application in animal production and the mitigation of the adverse effects of global warming.

## Additional Information and Declarations

### Data Availability

The following information was supplied regarding data availability: This is a review article; there is no raw data.
